# The Emergence of Selective Attention through Probabilistic Associations between Stimuli and Actions

**DOI:** 10.1371/journal.pone.0166174

**Published:** 2016-11-15

**Authors:** Luca Simione, Stefano Nolfi

**Affiliations:** Institute of Cognitive Sciences and Technologies, CNR, Rome, Italy; University of Verona, ITALY

## Abstract

In this paper we show how a multilayer neural network trained to master a context-dependent task in which the action co-varies with a certain stimulus in a first context and with a second stimulus in an alternative context exhibits selective attention, i.e. filtering out of irrelevant information. This effect is rather robust and it is observed in several variations of the experiment in which the characteristics of the network as well as of the training procedure have been varied. Our result demonstrates how the filtering out of irrelevant information can originate spontaneously as a consequence of the regularities present in context-dependent training set and therefore does not necessarily depend on specific architectural constraints. The post-evaluation of the network in an instructed-delay experimental scenario shows how the behaviour of the network is consistent with the data collected in neuropsychological studies. The analysis of the network at the end of the training process indicates how selective attention originates as a result of the effects caused by relevant and irrelevant stimuli mediated by context-dependent and context-independent bidirectional associations between stimuli and actions that are extracted by the network during the learning.

## Introduction

Selective attention can be defined as the capability to enhance the representations of stimuli that are relevant for the organism and to filter out unwanted perceptual information. It is a key process that is at the basis of goal-directed behaviour, object recognition, and memory storage. As suggested by the experimental findings to date, selective attention does not necessarily originate from dedicated cognitive processes (e.g. a process that operates by sequentially scanning the items present in the visual field, as proposed by Treisman and Gelade [1], or neural circuits, and might simply be the result of parallel interactions, occurring at different processing levels, between representations that compete for motor control and/or processing capacity, as in the Biased Competition model originally proposed by Desimone and Duncan [2– 4].

A first body of findings comes from visual search experiments, in which subjects, exposed to very brief presentation of two-dimensional stimuli (typically alphanumeric characters or geometrical shapes), are asked to recognise the presence or absence of a specific item, the target, presented among others, the distracters, by pressing alternative buttons or by verbally reporting the observed items. Usually in these experiments the target is defined on the basis of its features, such as the colour or the shape. Subjects’ response is very fast and accurate when the target is defined by a unique feature, producing the so-called ‘pop-out’ effect [5], otherwise the time increases with the number of distracters and the similarity between the visual features of the target and the distracters [1]. These results, originally used to sustain a serial deployment of a visual attention ‘spotlight’, have been later explained by a parallel interactive process. In particular, Bichot, Rossi and Desimone [6] showed how a feature-selection mechanism enhances the activity of the neurons that code for the relevant feature in parallel throughout the visual field, and thus that representations of distracters sharing one or more features with the target are enhanced as well, slowing down the response decision process. Overall, the results of these experiments indicate that representations of relevant objects are enhanced with respect to non-relevant information in terms of visually driven responses, at level of both individual neural responses and coherent firing within the critical neuronal population [7].

Other than enhancing the neural representations of the relevant items [8], selective attention is regarded to cause the suppression of irrelevant information, as pointed out by a further set of experiments in which subjects are requested to reach a target object in a 3D space, in the presence of similar distracting objects [9–15]. In this context, competition originates because only one object at the time can be reached and grasped, and consequently only the representation of the object to be reached is relevant to execute the appropriate response. Indeed, the results of these experiments indicate that when attention is focused on the target object, the presence of the distracters does not affect the trajectory with which the target is reached, irrespectively of the hand-distracter distance [13] and of the target-distracter similarity [9, 16]. On the contrary, when attention is also focused on a distracter, its features interfere with those of the target causing a deterioration of the action [16].

Concurring data have been collected in a series of related experiments in which primate subjects were performing an instructed-delay reaching task [17]. The task involved three phases during which the monkeys, located in front of a monitor, were exposed to: (a) two circles, one red and one blue, located around a fixation point, (b) a non-spatial cue indicating the colour of the target circle, and (c) an arrow pointer. After the presentation of the arrow pointer that acted as a ‘go’ signal, the monkeys were asked to move the pointer with a handle toward the location of the target circle. By recording the state of directionally tuned cells in the premotor cortex the authors observed two simultaneous sustained signals corresponding to the two reaching options during the first phase. Moreover they observed how the neural activity associated with the target and non-target circles increased and decreased respectively during the second phase, after the presentation of the cue indicating the colour of the target circle. Overall these data suggest that the conflict between the alternative actions is resolved through a competition process that operates at the pre-motor level and that is biased by the contextual cue indicating the object to be reached [18].

Previous theoretical and computational models postulate that the enhancement and the filtering out relevant and irrelevant stimuli originate as a result of architectural constraints that create a competition between the representation of alternative stimuli and/or actions [3, 19–23]. Such constraints consist of winner-takes-all circuits [3], mutual excitatory and inhibitory connections between neurons with similar and different receptive fields, respectively [19], lateral inhibitory connections between neurons of the same layers [20–23], or regulatory/gating mechanisms that block irrelevant information [24, 25].

In this paper we show how an artificial neural network trained for the ability to display context-dependent behaviours displays selective attention, i.e. represent the stimuli that are relevant for the action more accurately than the others, although they have been trained for re-generating all the stimuli. This effect is robust and does not depend on the specific characteristics of the neural architecture, or of the training set.

The obtained results demonstrate for the first time, to our knowledge, that selective attention can emerge in a neural network model as a result of the regularities present in the training data. It does not necessarily depend on the mutual inhibitory connections between representation of alternative stimuli or actions and/or on gating mechanisms that block the elaboration of irrelevant stimuli included in previous neural network models [3, 19, 20, 26].

More specifically, our results indicate that selective attention can originate simply as a result of the extraction during learning of associative rules that incorporate the bi-directional context-dependent and context-independent correlations between stimuli and actions. The reduced reconstruction of the irrelevant stimuli, in fact, originates as a result of the interference between alternative stimuli and actions mediated by these context-dependent and independent rules.

Finally, the post-evaluation of the behaviour of our network in an instructed-delay experimental scenario analogous to that investigated by Cisek and Kalaska [17] and Pastor-Bernier and Cisek [27] demonstrates that the behaviour of the network is consistent with the data collected on experimental trials with monkeys.

## Methods

As described in the Introduction, the need to select between different stimuli, or actions afforded by alternative stimuli, originates in context dependent situations in which the same stimuli might be relevant or irrelevant in different contexts. Thus, a minimal scenario that requires the development of a selective attention capability involves two stimuli (sA and sB), two contexts (cA and cB), and an action that co-varies with the stimulus sA in a context cA and with stimulus sB in the context cB. In each context, therefore, the features of one stimulus (sA or sB) are relevant for the selection of the correct action while the features of the other stimulus are irrelevant.

This might occur, for example, during the reaching and grasping in a 3D space of an object that is located near another “distracting” or not relevant object, for example grasping an apple placed near to an orange [10]. In this scenario, the context might be constituted, for example, by the sentence “could you please pass me the apple?”, indicating the object to grasp. The visual stimuli might be constituted by two objects of different colours detected through visual sensors. The context dependent action can consist in the appropriate reaching and grasping action. The characteristics of such action, i.e. the reaching trajectory and the aperture of the hand, should depend on the features of the relevant stimulus (the apple) and should not depend on the features of the irrelevant stimulus (the orange).

For this reason we set up a set of simulations in which a multi-layer neural network was exposed to a series of inputs organised as described above. More specifically, the neural network was exposed to a red and/or a green square object (sA and sB), a contextual stimulus indicating either the first or the second stimulus as relevant (respectively, cA or cB), and a target action directed toward the location of the relevant object (i.e. the location of the red object in context cA and the location of the green object in context cB).

### Neural network

We used a deep neural network model [28, 29], i.e. an artificial neural network model that exploits multiple layers of hidden units to build hierarchical internal representations of data. These networks are appealing since they include both bottom-up and top-down connections that enable them to recognize and to generate data and since, unlike back-propagation networks [30], they can learn efficiently multiple levels of representations [28].

**Fig 1:**
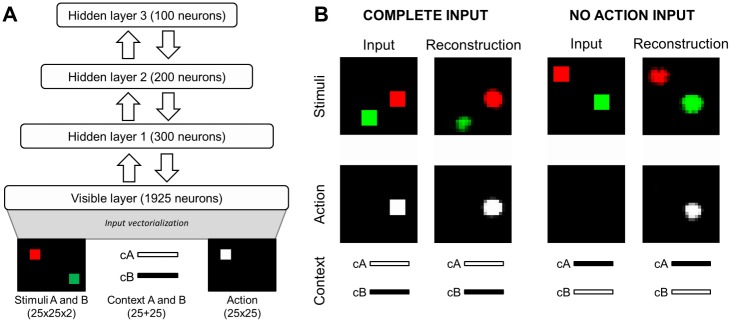
Neural network architecture and input patterns. (A, top) The neural network architecture included a visible layer and three hierarchically organised hidden layers. The network layers were fully connected through both bottom-up (upward arrows) and top-down (downward arrows) weights. (A, bottom) An example of input pattern including the visual input, with both the red and the green square presented, the context input, with cA input set to 1 (white) and cB input set to 0 (black), and the action input, with the white square presented in the same location of the stimulus that was relevant in the current context. (B) Example of inputs and corresponding reconstructions for a complete input pattern including both a red and green stimulus experienced in context cA (“Complete input”) and for an input pattern including both a red and green stimulus experienced in context cB in a test condition in which the action was not provided as input (“No action input”).

The architecture of our neural network included one visible layer and three hierarchically organized hidden layers, with each couple of layers connected through bottom-up (recognition) and top-down (generative) weights. The visible layer included two sets of red and green units arranged in two 25x25 matrices, enabling the detection of the presence and of the position of red and green stimuli, two vectors of 25 units enabling the detection of the current context (cA or cB) that consequently were activated in a mutually exclusive manner, and one matrix of 25x25 units indicating the action toward the target location (i.e. the location of the stimulus that was relevant in the current context). We used 25 units to encode the context since we observed that using a single or few units would lead to poorer performance. Overall the visible layer included 1925 units (see Fig 1, bottom). The first, second, and third hidden layers included respectively 300, 200 and 100 neurons. Neurons of adjacent layers were fully connected (see Fig 1, Top).

The neural network could be conveniently reduced to a stack of Restricted Boltzmann Machines (RBM), one for each hidden layer [31]. Each RBM has a layer of feature detectors (hidden units) *h*_*j*_ receiving weighted input *x*_*j*_ *= ∑w*_*ij*_*v*_*i*_ from the previous layer. The units in the same layer were not connected. The activation of each unit in the feature detector was computed by passing the input through the logistic function *h*_*j*_ *= 1/(1+e*^*−xj*^*)*. For more details on the neural network model, see the Appendix A.

### Learning

The learning process was realized through the contrastive divergence algorithm (CD) described in Hinton [28]. In accordance with the CD algorithm, the neural network was trained to generate the sensory data, i.e. maximizing the likelihood of reconstructing the input data.

A deep neural network, also called a deep belief network [32], is constituted by a stack of Restricted Boltzmann Machines (RBM). Each RBM is constituted by two layers of units (the visible and the hidden layer) fully connected via bidirectional weights. As reported above, the network does not include connections among the units of the same layer but only among units of different layers. For each input to the visible layer, the CD algorithm computes: (i) the state of the hidden layer units via the bottom-up weights (data-driven or positive phase), (ii) the new state of the visible layer units via the top-down weights, and (iii) the new state of the hidden layer units (model-driven or negative phase). The connection weights and biases are updated so to minimize the difference between the original and the regenerated state of the visible layer units. The next layers of hidden units are updated and trained sequentially in the same manner by using the previous layer of hidden units as the visible layer. Overall this means that deep neural networks are generative models of the training data that operate on the basis of a stuck of layers interconnected through bidirectional weights [33].

The training was performed for 500 epochs each including 5000 input patterns. For more details see the Appendix A.

### The training dataset

Each item included 1925 bits that specified the state of the corresponding visible units (that could assume either a 1.0 or 0.0 activation state). The red and green squares were represented by 5x5 units within the corresponding 25x25 matrix of units; the locations of the stimuli were selected randomly while ensuring that they did not overlap. The context was represented by two vectors of 25 units. The correct action was represented through the activation of 5x5 units within the corresponding matrix of 25x25 units. The specific units were selected on the basis of the current context and of the position of the relevant stimulus (see Fig 1).

The training dataset included a total of 5000 input patterns. Half of the patterns included only one visual stimulus (i.e. the irrelevant stimulus was absent), the corresponding action, and the corresponding context, whereas the other half of the input patterns included both a red and a green visual stimulus, with the correct action determined on the basis of the contextual input indicating the target colour. Data were generated so that the patterns including one or two stimuli and the patterns including the first or the second contexts were represented in the same number.

### Replications and analyses

Each experiment was replicated ten times, i.e. for each experimental condition we trained ten neural networks initialized with different randomly generated connection weights. After the training, to analyse the quality of data reconstruction we computed the network reconstructions of new input patterns (test data) containing both the red and green stimuli placed in randomly selected locations, in context cA as well as in context cB. The test data included 1000 input patterns for each testing condition. Half of such input patterns were generated to include an action toward the location of the red stimulus, while the other half included an action toward the location of the green stimulus. When specified in the text, the test data did not include the action input. For each testing condition, we computed for each replication the average activation of the units coding for the spatial locations of the two stimuli and of their corresponding actions, as well as the average activation of the units coding for the two contexts. Then, we computed the average values over all the ten replications and, when possible, we applied statistical analyses over the data collected by means of t-tests, considering the different replications of the neural network as different individuals.

## Results

In this section we describe the results obtained by analysing the behaviour of the neural network at the end of the training process and the characteristics of the data generated by the network when it was exposed to the same type of stimuli experienced during the training process, as well as to input patterns that were manipulated to verify the generalization capabilities of the network (see Fig 1B). To analyse the quality of data reconstruction, we computed the network reconstructions of input patterns containing both the stimuli placed in randomly selected locations, in context cA as well as in context cB. For each testing condition and for each replication, we computed the average activation of the units coding for the spatial locations of the two stimuli and of their corresponding actions, as well as the average activation of the units coding for the two contexts. Notice that, after a preliminary assessment of the capacity of the neural network reconstructions after the training, we used input patterns without the action input to test the generalization capability of the network.

To identify the role played by the different layers, we analysed how the output generated by the network varied when only the first or the first two layers were allowed to operate. Moreover, we describe how the output of the network varied over time when it was allowed to operate for a series of time steps on the basis of its self-generated state. Then, we present the result of an additional simulation in which the conditional and action information was provided before or after the sA and sB stimuli. Finally, we analysed the neural network internal structure in terms of weights and receptive fields of units, in order to assess how the network encoded the different information and the computational properties of the different layers.

### Quality of data reconstruction and generalization

**Fig 2:**
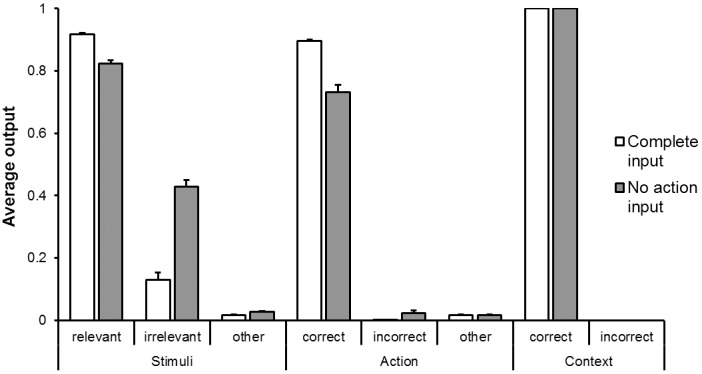
Average output activation of the units coding for stimuli, actions, and context in the data generated by the network. Data are shown separately for the units encoding the location of relevant and irrelevant stimuli, for the units encoding the correct and incorrect actions, for the units encoding the correct and incorrect context, and for the remaining units. The results obtained in a normal condition in which the network experienced input patterns including both the red and green stimuli are reported in white, and the results obtained in a test condition in which the network experienced the same items but in which the activation of all action units was set to 0.0 are reported in grey.

As expected, with complete input patterns the network tended to reconstruct accurately all the three types of information (stimuli, context, and action), as shown by the very low activation of the units that coded for locations with no stimulus or action (see Fig 1B for a typical case). However, the network reconstructed the relevant stimulus (M = 0.92) better than the irrelevant one (M = 0.13), p <.001, despite the training algorithm attempts to maximize the reconstruction of all information (see Fig 2). These results indicated that the network extracted the functional relationships between the three input channels and used such relationships to strengthen the activation of the relevant stimulus and/or to inhibit the activation of the irrelevant one.

The acquisition of the relationships between the three input channels (stimuli, contexts, and actions) enabled the network to generalize by self-generating missing information. To verify the generalization ability of the trained network, we evaluated it in a test condition in which the stimuli and the contextual information were provided as usual while the action input was not provided (i.e. the state of all action units was set to 0.0). In this condition (see Fig 2) the network was capable of re-generating not only the information that was directly provided but also the correct action (M = 0.73). Notice how the network also tended to erroneously generate a second incorrect action, targeted toward the position of the irrelevant stimulus. In fact, in this condition the incorrect action location (M = 0.024) was more activated than the other locations (M = 0.017), p <.001, whereas with the complete input patterns the incorrect action location (M = 0.0005) was less activated than the other action locations (M = 0.02), p <.001. Then, by comparing activations of correct and incorrect actions in the condition with no action input with the complete condition, we observed that correct actions was less activated while incorrect actions was more activated when the action input was missing, ps <.01 in both cases. Moreover, the accuracy of the target stimulus reconstruction (M = 0.82) and the relative inhibition of the non-target stimulus (M = 0.43) were less marked with respect to the normal condition, all ps <.01.

Overall these results indicated that the data generated by the network were influenced in a significant way by both bottom-up and top-down information, corresponding respectively to the input data and to the regularities extracted by the network that encoded how the information provided in the three channels co-varied. We will come back on the interpretation of these results in the last part of the Results section.

### Control simulations

To verify whether the results described in the previous section depended on the training algorithm, on the parameters used, and/or on the complexity of the task we ran several control experiments that are reported in details in Appendix B. In these experiments we manipulated all the critical parameters of the model [34] and the characteristics of the training set.

In these control experiments, we varied the usage of the units' drop-out during the learning (see second section of the Appendix A), the duration of the training procedure, the presence of patterns including single stimuli in the training set, the size of the network, the architecture of the network, and the variability of stimuli in space. The neural networks trained in these control conditions were evaluated by using complete input patterns.

Remarkably we obtained qualitatively similar results in all experimental conditions with the exception of the condition in which the stimuli could assume only two alternative locations, i.e. the red and green stimuli were always placed in either the top-left or the bottom-right location. Indeed, only in this case the quality of the reconstruction of the irrelevant stimuli did not differ significantly from that of the relevant stimuli (see Appendix B).

The fact that the effect disappeared in the simplified scenario could be explained by considering that the interference that occurs between relevant and irrelevant stimuli and action depends on the level of variation of the locations of the stimuli and on level of overlap between stimuli in sensory space.

In some conditions, i.e. when the networks experienced only double stimuli patterns or in which the size and the architecture of the network was varied, the difference between the reconstruction level of the relevant and irrelevant stimuli varied. However, also in these cases the level of activation of irrelevant stimuli was significantly lower than the level of activations of relevant stimuli. This result shows that some parameters of the training could influence the magnitude of the 'attentional' effect but also that this effect was robust with reference to a variety of conditions and training procedures.

### On the roles of the different processing layers

To verify the role played by the different internal layers, we analysed the reconstructions generated by the network in three test conditions in which only the first or only the first two or all three layers of internal neurons were allowed to operate and in which the action input was not provided.

**Fig 3:**
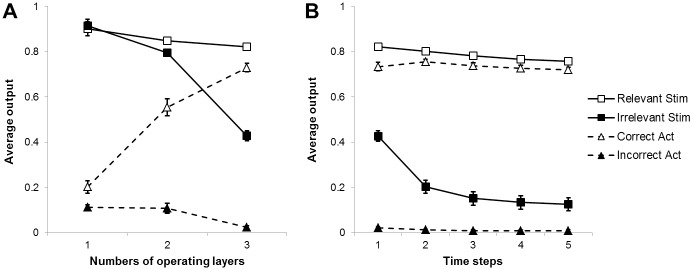
The iterative solution developed by the neural network. (A) Data generated by the network in three test conditions in which only the first, or only the first two, or all three hidden layers were allowed to operate (1, 2, and 3, respectively). (B) Data generated by the network after one to five processing time steps in which the network operated on the basis of its previously self-generated output.

As shown in Fig 3, panel A, when only the first internal layer was allowed to operate, the two stimuli were reconstructed equally well (M_Relevant_ = 0.9, M_Irrelevant_ = 0.91, p = .34), whereas they significantly differed when also the second (M_Relevant_ = 0.85, M_Irrelevant_ = 0.79, p <.001), and the third hidden layer (M_Relevant_ = 0.82, M_Irrelevant_ = 0.43, p <.001) were allowed to operate. The analysis of the action reconstruction revealed that, when only the first internal layer was allowed to operate, the correct action was partially activated (M = 0.2), and the incorrect action was partially activated as well (M = 0.11), although less than the correct action, p <.001. The difference in activation between the correct and incorrect action increased as the second (M_Correct_ = 0.55, M_Incorrect_ = 0.11, p <.001) and the third (M_Correct_ = 0.73, M_Incorrect_ = 0.02, p <.001) internal layers were also involved.

This pattern of results suggests that the network extracted from its learning experiences two types of associative rules: (i) context-independent associations between stimuli and actions (originating from the fact that a stimulus in a given position was often, although not always, associated with an action targeted toward that location and vice versa an action was often, although not always, associated to the presence of a stimulus in the same relative location), and (ii) context-dependent associations between stimuli, contexts, and actions (i.e. originating from the fact that the presence of a stimulus in a given location was always associated with the experience of an action targeted toward that location in the corresponding context). This explanation was further supported by the analysis reported in the last part of the Results section.

Moreover, the differences between the activation of the relevant and irrelevant stimuli and between the activation of the correct and incorrect actions become progressively larger as a result of the contribution of the second and of the third internal layers (Fig 3, panel A). This suggests that the data generated by the network were the results of the iterated application of the same type of context-independent and dependent associative rules during the three successive processing stages.

The iterative nature of the solution developed by the network (that consisted in applying the same rules over and over again within the successive processing layers) was further demonstrated by a series of tests in which the network, after being exposed to the input pattern, was then allowed to operate on the basis of its self-generated input for four additional processing time steps (see Fig 3B). Indeed the reconstruction of the irrelevant stimulus further decreased from the first to the second time step (M_1_ = 0.43, M_2_ = 0.2, p <.001), and from the second to the third step (M_2_ = 0.2, M_3_ = 0.15, p <.001). The states reconstructed during succeeding steps were not statistically different from the states reconstructed at step 3.

### Instructed-delay experiments

In the additional tests reported in this section we analysed the behaviour of the network in situations in which the presentation of the contextual information or of the stimuli was anticipated or delayed in time. The motivation behind these additional analyses was that they enabled us to compare the behaviour of the neural network in an experimental scenario analogous to the instructed-delay experimental scenario investigated by Cisek and Kalaska [17] and Pastor-Bernier and Cisek [27] with monkeys. In these experiments the monkeys were rewarded for “reaching” one of two alternative stimuli displayed with different colours on a computer screen by moving the computer pointer through a joystick. The contextual information encoding the target to be reached was provided by a cue painted in one of two possible colours, and it was experienced either before or after the presentation of the two stimuli. By monitoring the activation state of the monkeys’ premotor cortex, Cisek and collaborators observed that the presentation of the two stimuli before the contextual information led to the activation of the premotor neurons encoding either the action appropriate for reaching the target stimulus or the action appropriate for reaching the irrelevant stimulus, and to a rapid suppression of the latter activity following the presentation of the contextual information. Instead, when the contextual information was presented before the presentation of the two visual stimuli, only the activation of the premotor neurons encoding the action toward the relevant stimulus was observed.

**Fig 4:**
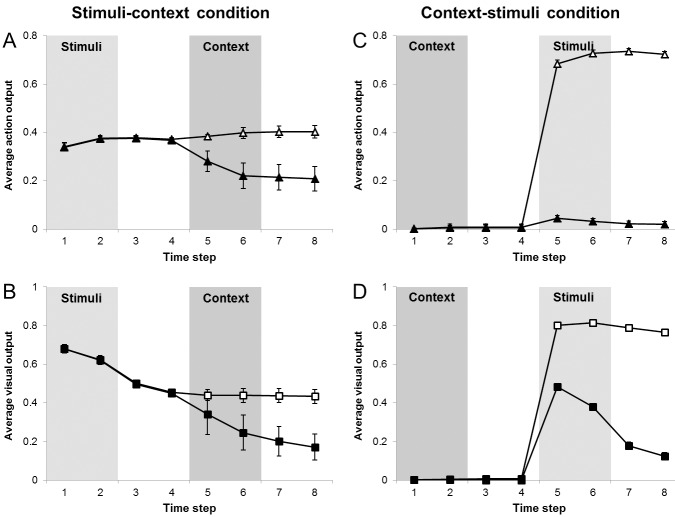
Average activation of the units encoding the relevant and the irrelevant stimulus as well as the correct and the incorrect action during eight successive processing steps. The processing steps in which the stimuli and the contextual information was provided are indicated on the top of each panel. (A) Experimental condition in which the presentation of the stimuli preceded the presentation of the context. (B) Experimental condition in which the presentation of the context preceded the presentation of the stimuli.

Thus, we analysed the behaviour of our network at the end of the training in two experimental conditions. In the first condition (Fig 4A), the network experienced the two stimuli (sA and sB) first, followed by a blank (a period in which the network did not receive any input), and then it experienced the contextual stimulus (cA or cB) followed by a second blank period. In the second condition (Fig 4B), the network experienced the contextual stimulus (cA or cB), a blank period, the stimuli (sA and sB), and finally another blank period. In both experimental conditions the action input was never provided (i.e. the external activation of all action units was set to 0). To enable the neural network to process information over time, we allowed the network to operate for eight consecutive time steps Each stimulus or blank period was presented for two subsequent time steps, i.e. the first stimulus was presented at times one and two, no stimulus was presented at times three and four, the second stimulus was presented at times five and six, followed by another blank period at time seven and eight. During each time step, we computed the network reconstruction of an input pattern composed by mixing the externally provided information for 9/10 and the reconstruction computed by the network during the previous time step for 1/10 (with the exception of the first time step in which the input was completely externally determined).

Consistently with what has been observed by Cisek and collaborators on monkeys, in the condition in which the stimuli were provided before the contextual information, the correct and incorrect actions (Fig 4C) reached a similar activation value during the first four time steps, in which no contextual information was provided (M_Correct_ = [0.34, 0.38, 0.38, 0.37], M_Incorrect_ = [0.34, 0.37, 0.38, 0.37], ps>.42), and then rapidly differentiated from the fifth time step on, when the network experienced the context (M_Correct_ = [0.38, 0.4, 0.4, 0.4], M_Incorrect_ = [0.28, 0.22, 0.21, 0.2], p <.01 in all cases). Instead, in the condition in which the contextual information was provided before the stimuli, the two alternative actions became activated only from time step 5 on, when the network experienced the stimuli (Fig 4D), with an immediate advantage for the action toward the relevant stimulus (M_Correct_ = [0.68, 0.73, 0.74, 0.72], M_Incorrect_ = [0.04, 0.03, 0.02, 0.02], p <.01 in all cases). A similar pattern of results was observed for the differential activation of the relevant and the irrelevant stimuli in the two experimental conditions (Fig 4E and 4F).

Notice however that in the former condition, in which the stimuli were provided before the contextual information, the level of activation of the action targeted toward the relevant stimulus did not increase. The difference between the activation of the two actions increased only as a result of the reduced activation of the action targeted toward the irrelevant stimulus. Interestingly, this suggests that the network did not extracted strong mutual inhibitory associations between alternative actions as a result of learning.

### Network analysis

**Fig 5:**
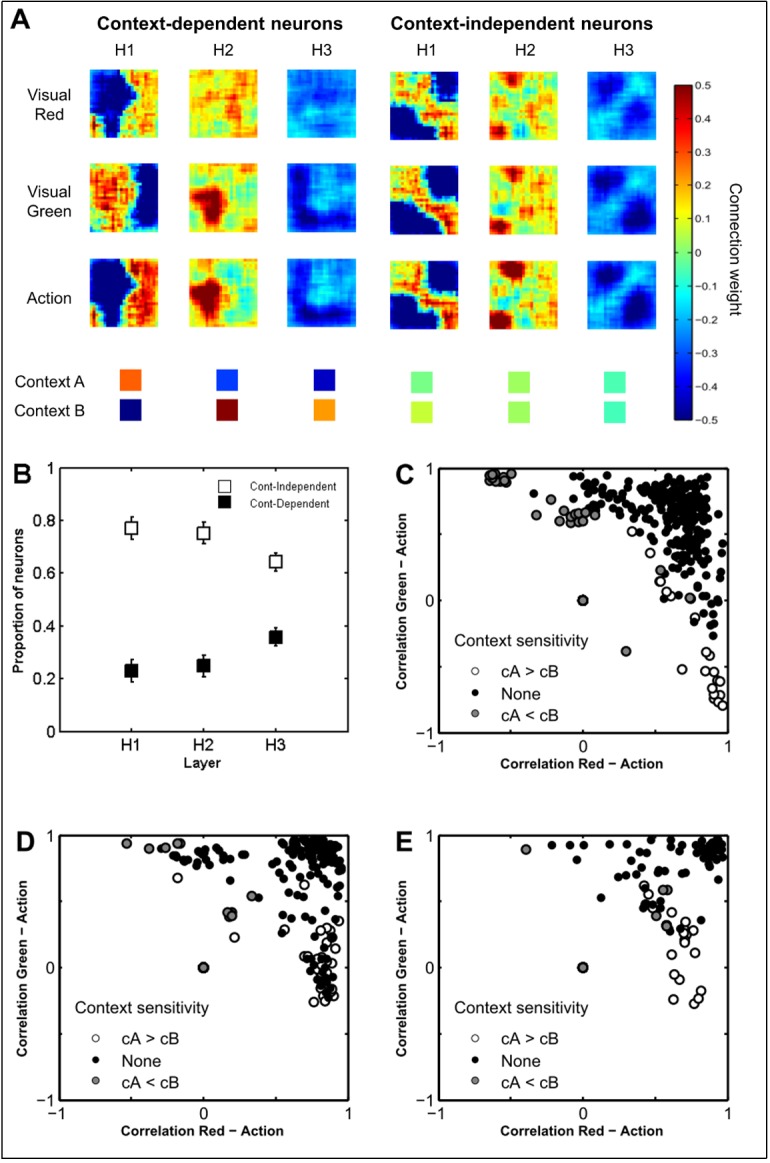
Receptive fields of the network's neurons. (A) Receptive fields of representative neurons in the first (H1), second (H2), and third (H3) hidden layers, belonging to the context-dependent and context-independent classes, respectively. Panel B: average proportion of context-dependent and context-independent neurons in the three hidden layers. (C-E) Correlations between the receptive fields of the two visual inputs and the action input computed for each neuron in the first (C), second (D), and third (E) hidden layer of the first replication of the neural network. Each data point represents a neuron and is depicted in a colour that indicated whether it is sensitive to context A or B, or none.

In this section we present the analysis of the neural network connections in order to understand how they encoded the inputs. To this aim, we computed the receptive fields of the neurons of the first, second, and third hidden layers of the neural network. To analyse the role of the individual neurons of the first layer, we generated 2D images of the neurons’ receptive fields that visualize with pixels of different colours the strengths of each neuron's incoming connections. Receptive fields depicted in the Fig 5 were drawn from a single replication (other replications led to similar results; data were not shown for reason of space). To enhance readability, for each neuron we generated 5 pictures displaying the strengths of the connections coming from the two matrices of 25x25 red and green visual receptors, from the 25x25 matrix of units encoding the action, and from the two sets of 25 units encoding the context. To compute the receptive fields of neurons of the second and third layers, we used a linear combination of the weight matrices of the lower layers, imposing a threshold of θ = 0.5 on the absolute values of the weights in order to select only the stronger connections [29].

Fig 5A shows the receptive fields of neurons sampled from the different layers of the neural network. Incoming excitatory connections are depicted from yellow to red, whereas inhibitory connections are depicted from light to dark blue, according to their strength. The visual inspection of these pictures indicates that, in all layers, neurons encoded context-independent or context-dependent regularities concerning specific selected locations (see Fig 5A). Context-independent neurons were excited by stimuli and actions presented in selected locations, independently from the context and from the colour of the stimuli, and were inhibited by the stimuli and actions experienced in other locations. For example, the context-independent neuron of the first layer shown in Fig 5A was excited by stimuli and actions occurring in the top-left, central, and bottom-right locations whereas it was inhibited by stimuli and actions occurring in other locations, and was almost equally excited by contexts cA and cB. Context-dependent neurons, instead, were excited and inhibited respectively by coherent and incoherent visual, action, and contextual information, showing complementary receptive fields for visual input of the two colours. For example, as shown in Fig 5A, the reported context-dependent neuron of the first layer was strongly excited by context cA and strongly inhibited by context cB, was excited by red stimuli and actions located on the right side, and it was inhibited by green stimuli located on the right side. The same pattern applied to neurons in all layers, with context-independent neurons displaying similar receptive fields for both colours and actions and no sensitivity to context. Instead, context-dependent neurons showed high sensitivity to the context, complementary receptive fields for the locations of red and green objects, and a receptive field for the action superimposable to that of the colour relevant in the context in which they were activated.

All neurons of our neural network tended to belong either to the first or to the second type. This confirms the hypothesis outlined above that the network operated by extracting and by using context-dependent and context-independent regularities. To identify whether a neuron belonged to the first or the second class, we computed for each neuron the sensitivity to contextual information and the correlation between the weights encoding the location of the action and that encoding either the red or the green visual stimulus (see Fig 5C, 5D and 5E). As shown in Fig 5C, 5D and 5E, the majority of the neurons belonged to the context-independent class that was characterized by neurons in which the absolute difference between the mean of the connection weights encoding one context and the mean of the connection weights encoding the other context was lower than θ = 0.3 (‘Context sensitivity’ = ‘None’). Neurons of this former class were also characterized by a positive correlation between the weights encoding the position of the action and that encoding the position of both the red and the green visual stimuli. The remaining neurons belonged to the context-dependent class and were characterized by greater sensitivity to the cA context with respect to the cB context (‘cA > cB’), or vice versa (‘cB > cA’). Neurons of this latter class were also characterized by a positive correlation between the weights encoding the location of the action and those encoding visual stimuli of the colour indicated by the corresponding context, and by a negative correlation between the weights encoding the location of the action and those encoding the visual stimuli of the other colour.

Remarkably, we found these two types of neurons in all the three layers (Fig 5B), confirming the hypothesis that the three layers performed a similar computation. However the proportion of context-dependent neurons increased from the second to the third layer (M_2_ = 0.25, M_3_ = 0.39, p <.001) and thus the percentage of context-independent neurons decreased from the second to the third layer (M_2_ = 0.75, M_3_ = 0.61, p <.001). The numbers of context-dependent and independent neurons in the first and second layer did not differ statistically.

**Fig 6:**
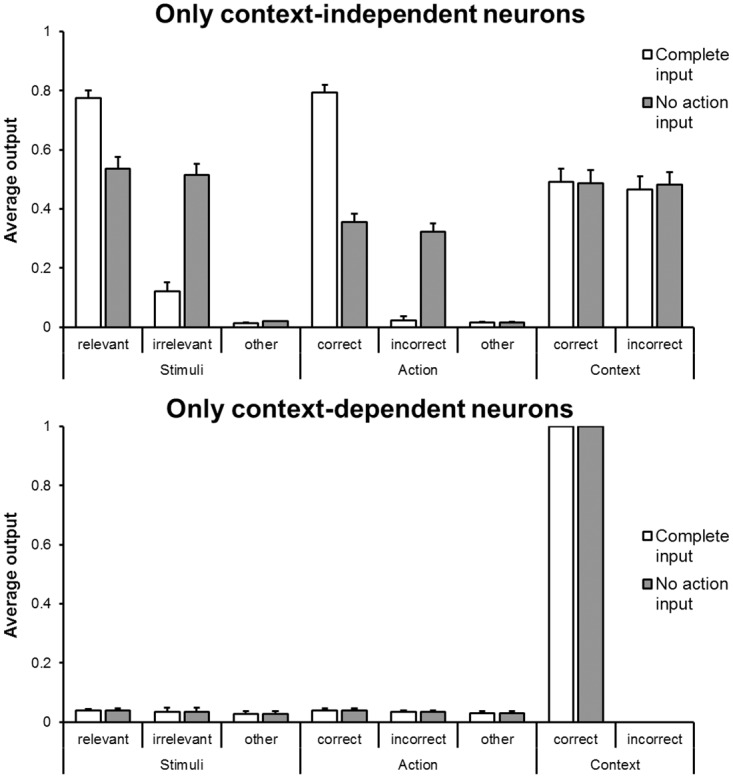
On the role of context-independent and context-dependent neurons. Average output activation of the units coding for stimuli, actions, and context in control condition in which only the context-independent neurons and the context-dependent neurons was allowed to operate (upper and lower panel, respectively).

To verify the effective computation carried out by each neural population, we performed an analysis of the behaviour of the network in a control condition in which only the context-independent or the -dependent neurons were allowed to operate (Fig 6). The analysis of the first condition (Fig 6, upper panel) indicated that the relevant stimuli were reconstructed better than the irrelevant ones in the case of complete input patterns (M_relevant_ = 0.77, M_irrelevant_ = 0.12, p <.001) while they were reconstructed equally well in the no-action case (M_relevant_ = 0.53, M_irrelevant_ = 0.51, p = .43). The correct action was reconstructed better than the wrong action in the case of complete input patterns (M_correct_ = 0.79, M_wrong_ = 0.02, p <.001) while they were reconstructed equally well in the no-action condition (M_correct_ = 0.35, M_wrong_ = 0.12, p = .09). Finally, the activations of the two contexts did not differ in both complete (M_correct_ = 0.49, M_wrong_ = 0.46, p = .21) and no-action conditions (M_correct_ = 0.49, M_wrong_ = 0.48, p = .79).

Instead, the analysis of the behaviour of the network in the control condition in which only the context-dependent neurons were allowed to operate (Fig 6, lower panel) indicated that only the reconstructions of the correct and wrong contexts were correct and statistically different (M_correct_ = 1.00, M_wrong_ = 0.00, p <.005) in both complete and no-action conditions, whereas the activation of the relevant and irrelevant stimuli as well as of the correct and wrong actions were very low and not statistically different in both complete and no-action conditions.

These results showed how the reconstructions of the stimuli and of the actions were realized by the context-independent neurons while the reconstructions of the contexts were realized by the context-dependent neurons. However, the context-dependent neurons also served to inhibit the wrong action which was partially activated by the context-independent neurons, as demonstrated by the higher activation of the incorrect action in the test condition without the action input (M = 0.32) in which the context-dependent neurons were removed (Fig 6, upper) respect to the complete condition (M = 0.02; see Fig 2), p <.001.

Finally, the fact that the filtering of the irrelevant stimuli was observed also in the control condition in which the context-dependent neurons were not allowed to operate demonstrates that it was primarily due to the context-independent neurons. More specifically, it could be explained by considering that context-independent neurons specialized for certain locations often tended to inhibit stimuli and actions presented in other locations (see Fig 5A) and that the context-independent neurons triggered by the relevant stimuli tended to be more activated than those triggered by the irrelevant ones (due to the fact that the former were excited by stimuli and actions located in the same positions while the latter were excited by the stimuli only). Then, when the action input was missing and thus the context-independent neurons encoding the two visual stimuli were equally excited, the network was not able to select the correct action with the context-independent neurons alone and it needed the contextual information encoded mostly by the context-dependent neurons in order to provide the regeneration of the correct action only.

The stimuli generated by the network also depends on the network input, as demonstrated by the weaker filtering out of the irrelevant stimulus and the increased activation of the wrong action obtained when the action was not provided as input (see Fig 2). Overall the obtained results indicate that the output produced by the network is the result of the interplay between the input received by the network and the bi-directional context-dependent and–-independent regularities extracted by the network. Bi-directionality refers to the fact that the context-independent associative rule between a stimulus located in a certain position and an action targeted toward the same position imply both that the activation of a stimulus located in a certain position enhances the activation of an action targeted toward the same position and that the activation of an action targeted toward a certain position tends to enhance the activation of a stimulus located in the same position.

## Discussion

In this paper we demonstrated how selective attention emerges spontaneously during the learning of context dependent behaviour. To verify this hypothesis we subjected a multilayer associative neural network to a series of training experiences in which the elicited action co-varies with a stimulus of a given colour in one context and with a stimulus of another colour in an alternative context.

The analysis of the behaviour of the network showed how the knowledge acquired during the training process enables it to re-generate the experienced items and to reconstruct missing information, for example to generate the correct action in a control condition in which the network received as input the stimuli and the contextual information but not the action. Moreover the analysis of the behaviour of the network at the end of the training process shows how it spontaneously produces a selective attention process. Indeed, in the data regenerated by the network the irrelevant stimulus is less activated with respect to the one that is relevant in the current context, although both stimuli are provided as input and although the network was trained for regenerating all input data (including the irrelevant stimulus). The differential reconstruction of the relevant and irrelevant stimuli appears to be a rather robust phenomenon since it is observed in a large variety of control conditions in which the size and architecture of the neural network, and the characteristics of the training data and of the training process have been varied.

The behaviour of the network in both a normal condition, in which all information is concurrently available, and in the instructed-delay condition, in which the presentation of the contextual information or of the stimuli was delayed in time, shows how the same network model accounts for different experimental results. In fact, both the results obtained in selective reaching experiments performed by Castiello and others [16] in which the representation of irrelevant stimuli tends to be filtered out at earlier processing stages, and the results showed by Cisek and Kalaska [17] on the instructed-delay task in which the information of all stimuli appeared to be processed in parallel up to the premotor stage before the presentation of the contextual information, could be explained by our neural model. The combined effects of context-independent and -dependent rules could contribute to reconcile the apparent contradiction, in the literature on affordance, between the findings that support an automatic and context-independent activation of the action afforded by experienced objects and the alternative findings that rather indicate how the activation of the action afforded by an object is determined conditionally by the context [35, 36].

The analysis of the internal organization of our network suggests that the emergence of a selective attention capability might originate as a result of the incorporation of the probabilistic regularities occurring between stimuli and actions. The assimilation of these regularities, in fact, leads to development of both context-dependent and context-independent neurons (that tended to trigger the elicitation of the action afforded by a stimulus independently from the context). As far as we know, this constitutes a new hypothesis on the mechanisms that can be at the basis of selective attention. As such, it might deserve the conduction of new experimental studies carried out to verify its explanatory validity for human and/or animal behaviour. In particular, in future works the role of context-dependent and -independent bidirectional associations between stimuli and action might be verified through behavioural and/or neurophysiological studies. A promising research area in that respect is constituted by visuo-motor coordination tasks. Indeed, in a recent study [37] the authors found two populations of context-independent and context-dependent in the mediotemporal (MT) lobe. The first neural population encodes information about the position, the speed, and the acceleration of the hand irrespectively from the context, i.e. the target of the action. The second group of neurons, instead, encodes the same information but in a specific visual context, with reference to specific visual objects.

Overall these results support the hypothesis that the ability to selectively attend stimuli, demonstrated by the differential regeneration of the relevant and the irrelevant ones, does not necessarily require the postulation of architectural constraints but can emerge simply as the result of the statistical regularities present in the learning experiences. This implies that the presence of the mutual inhibitory connections between neurons representing alternative stimuli and/or actions that characterize the neural networks models developed so far [3, 19, 20, 38] is not a necessary requirement.

Notice that our finding does not imply that the ability to enhance relevant information and/or filtering out irrelevant information is not influenced by the architecture of the brain. It only demonstrates that selective attention can occur also in the absence of specific architectural constraints. On the other hand, the fact that selective attention could originate simply as a result of the acquisition of the regularities present in the training data opens the possibility for alternative explanations of this phenomenon. Moreover, it can help us to explain why the tendency to filter out irrelevant information applies to rather diverse capabilities such as visual search and selective listening [39].

## Appendix A. Neural Network and Training Algorithm

### Neural network

We used a deep neural network model, i.e. a generative model that observe and model data by using local signals only, and in which learning corresponds to fit a generative model to the data [29]. The neural network consisted of a stack of Restricted Boltzmann Machines (RBM), one for each hidden layer [31]. The network included stochastic units, fully connected with symmetric weights and without self-connections, where each unit fires with a probability depending on the weighted sum of its inputs. Each RBM has a visible layer *v* and a layer of feature detectors (hidden units) *h*. The activation of the units in the visible layer consists in the vectorization of the input patterns for the first RBM and in the activation of the hidden layer of the previous RBM for the second and third layers. The activation of each unit in the feature detector was computed by passing the input through the logistic function:

$$h_{j}=1/1\left(+e^{-x_{j}-b_{j}}\right)$$

where *x*_*j*_ and *b*_*j*_ are respectively the weighted input from the visible layer and the bias of the *j-th* unit of the hidden layer. The receiving weighted input *x* is computed according to:

$$x_{j}=\sum w_{ij}v_{j}$$

The network’s connection weights were initially set randomly from a normal distribution with μ = 0.0 and σ = 0.1, and all the biases are set to 0.

### Learning process details

The learning procedure implemented for training the neural network was the contrastive divergence (CD) that works to minimize the difference between the data provided as input and the network reconstruction of the data. For each hidden layer, during the “positive phase”, given an input vector *v*_*i*_^+^ the activation of the feature detectors *h*_*j*_^+^ was first computed. Then, the state of the input vector *v*_*i*_^-^ is inferred from the state of the feature detectors *h*_*j*_ (“negative” phase). The weights *w*_*ij*_ were updated with a small learning fraction ε = 0.002 (for each input pattern) of the difference between input-output correlations measured in the positive and the negative phases:

$$\Delta w_{ji}=\epsilon \left(v_{i}^{+}h_{j}^{+}-v_{i}^{-}h_{j}^{-}\right)$$

The neuron biases were updated with the same learning rate.

To avoid over fitting, we applied the drop-out method [40], in which each hidden neuron had equal probability (p = 50%) to operate and thus being trained or to be dropped in each learning epoch.

## Appendix B. Control Simulations

In this section we describe in details the control simulations performed. Each condition was replicated 10 times. The reconstructions of the relevant and irrelevant stimuli (Table 1) differ significantly in all cases (p <.01) with the exception of the case with two locations only (p = .23).

**Table 1:** Average reconstruction of the relevant stimuli, irrelevant stimuli, correct action, and incorrect action in each control experiment.

Control condition	Relevant stimulus	Irrelevant stimulus	Correct action	Incorrect action
No single stim in training	0.89 *(0*.*003)*	0.63 *(0*.*038)*	0.87 *(0*.*006)*	0.003 *(0*.*001)*
Half learning epochs	0.85 *(0*.*019)*	0.07 *(0*.*009)*	0.82 *(0*.*022)*	0.001 *(0*.*001)*
Double learning epochs	0.91 *(0*.*005)*	0.15 *(0*.*072)*	0.89 *(0*.*009)*	0.001 *(0*.*0003)*
No drop-out	0.76 *(0*.*025)*	0.20 *(0*.*02)*	0.73 *(0*.*029)*	0.008 *(0*.*002)*
Two locations only	0.99 *(0*.*0001)*	0.98 *(0*.*05)*	1.00 *(0*.*00)*	0.000 *(0*.*0)*
Modular architecture	0.85 *(0*.*004)*	0.51 *(0*.*022)*	0.89 *(0*.*004)*	0.00001 *(0*.*0003)*
Half neurons	0.76 *(0*.*018)*	0.07 *(0*.*012)*	0.73 *(0*.*024)*	0.003 *(0*.*001)*
Double neurons	0.96 *(0*.*002)*	0.58 *(0*.*015)*	0.95 *(0*.*002)*	0.001 *(0*.*0003)*

Note. Data obtained from 10 replications of each experimental condition. The number between parentheses indicates the standard deviation. The reconstruction of the relevant and irrelevant stimuli significantly differ in all cases (p <.01) with the exception of the simulation with two locations only (p = .23).

### Variation in training set

To verify whether the results depended on the presence in the training set of both patterns including two stimuli and patterns including a single stimulus we carried out a control experiment in which we only included patterns with two stimuli in the training set.

### Variation in training duration

In two additional control simulations we varied the duration of the training period by using the half or the double of the training epochs with respect to our basic experiment (i.e. 250 and 1000 epochs, respectively).

### Not-usage of the drop-out

We carried out a control simulation in which we did not use the drop-out technique described in the Appendix A. In other words we used the original learning algorithm developed by Hinton [32]. Following the recommendation of Srivastava et al. [40], we used a learning rate ε = 0.0002 in this case.

### Usage of only two locations for the stimuli

In this control condition we trained and tested the neural network with input patterns in which the stimuli could be presented only in two non-overlapping locations, i.e. the red and green stimuli are always randomly placed in either the top-left or the bottom-right location.

### Variation in neural network architecture

In this control simulation we used a modular neural controller in which the units of the visible layer encoding the stimuli, the context, and the actions projected their connections to three corresponding blocks of neurons of the first layer (that included 150, 50, and 100 units, respectively), that then projected their connections to three corresponding blocks of neurons of the second layer (that included 100, 25, and 75 neurons, respectively). The neurons of the second layer, instead, were fully connected with the neurons of the third layer (that included 100 neurons). This type of architecture forces the network to first process visual, contextual, and action information separately.

### Variation of the number of internal neurons

In these control conditions, we varied the size of the internal layers. In particular in a first control condition we used half neurons (i.e. we provided the network with 150, 100, and 50 neurons respectively in the first, second, and third layer) while in a second control condition we doubled the number of neurons (i.e. we provided the network with 600, 400, and 200 neurons respectively in the first, second, and third layer).
